# Supplementary material for: Dynamic interplay between target search and recognition for a Type I CRISPR-Cas system
Source: Nat Commun. 2023 Jun 20;14:3654. doi: 10.1038/s41467-023-38790-1 (PMC10281945; doi:10.1038/s41467-023-38790-1)
Supplement: Supplementary file 1 — Supplementary Information [file 41467_2023_38790_MOESM1_ESM.pdf]

Supplementary Figures and Tables

Table S1. Samples Sizes *N* for Main Fig. 5 c-e.

		**Torque [pN nM]**	***N***
Panel c-e	T1	-4.883	29
		-3.958	117
		-4.201	113
		-4.436	61
		-4.663	53
	T2	-4.663	53
		-4.883	22
		-5.098	58
		-5.307	34
		-5.512	26
		-5.712	42
		-5.908	48
		-6.101	68
	T4	-6.477	21
		-6.841	16

Table S2. Oligonucleotides and plasmids used in the study.

Oligo ID	Description	Sequence
TS89	Cloning of Cse8e with C-terminal His-tag to pBAD24, forward Eco31I [NcoI tilt]	ggtctcagatccaatgagtcggtttaatttacttgatgaac
TS90	Cloning of Cse8e with C-terminal His-tag to pBAD24, reverse XhoI	ctcgagttttaactgttgacgaagccaaaaatcg
IR76	C252S and C262S mutagenesis of Cas8e, forward	gaatccggtaatgtagaaaatattcagcgaccttcctgggaacgtaaaag
IR77	C252S and C262S mutagenesis of Cas8e, reverse	cccaggaaggtcgctgaatattttctacattaccggattcattatacg
IR92	V76C mutagenesis of Cas6, forward	caaaagcttgaaaaatatggttgtgtaggaagcgctc
IR93	V76C mutagenesis of Cas6, reverse	cctacacaaccatatttttcaagcttttgtaaattagg
JM_Not1_forward	Forward primer for making construct for combined magnetic tweezers and fluorescence measurements	tattacgccagcggccgcaagggggatgtgctgcaag
PA_Cascade_Rev_SpeI_2.1	Reverse primer for making construct for combined magnetic tweezers and fluorescence measurements	gatcagttgggtgcacgagtggactagtgtgaa
PS1-T20f/r	Targets with AAN-PAM with 20 PAM-distal mismatches cloned into pUC19 for combined magnetic tweezers and fluorescence measurements	gaccaccctttttgatataatatacctatatcataccggagggtgcgtattcggcagatacgttctgagggaa
PS1-T20f/r-AG	Targets with AGN-PAM with 20 PAM-distal mismatches cloned into pUC19 for combined magnetic tweezers and fluorescence measurements	gaccaccctttttgatatagtatacctatatcataccggagggtgcgtattcggcagatacgttctgagggaa
PS1-T20f/r-CC	Targets with CCN-PAM with 20 PAM-distal mismatches cloned into pUC19 for combined magnetic tweezers and fluorescence measurements	gaccaccctttttgatatcctatacctatatcataccggagggtgcgtattcggcagatacgttctgagggaa
PS1-T1f	Targets with one PAM-distal mismatch for target recognition measurements	gaccaccctttttgatataatatacctatatcaatggcctcccacgcataagggcagatacgttctgagggaa
PS1-T2f	Targets with two PAM-distal mismatches for target recognition measurements	gaccaccctttttgatataatatacctatatcaatggcctcccacgcataacggcagatacgttctgagggaa
PS1-T4f	Targets with four PAM-distal mismatches for target recognition measurements	gaccaccctttttgatataatatacctatatcaatggcctcccacgcatttcggcagatacgttctgagggaa
PI_ZT_PNL_F	Matching target oligo for EMSA	ggaccacgcataatatacctatatcaatggcctcccacgcataagcagtg
Nonmatching-F	Non-matching target oligo for EMSA	cggaccacccataagctgtctttcgctgctgagggtacg
**Plasmid**	**Description**	**Reference**
**pUC19**	Plasmid used for cloning plasmids with different CRISPR targets	New England Biolabs, #N3041S
pCRh	Contains homogeneous CRISPR region with 6 identical spacers	Sinkunas et al., 2013[1]
pCDF-Cascade-Cas8e-C252S-C262S-Cas6-V76C	Plasmid encoding double mutant of Cas8e C252S-C262S and a mutant of Cas6 V76C	This study
pBAD24-CHis	Used for cloning of Cas8e fusing with C-terminal His_6-tag	Thermo Fisher Scientific
pBAD-Cas7-C-His	Contains Cas7 protein with C-terminal His_6-tag under *araC* based promoter.	Sinkunas et al., 2013[1]
pCas3	Contains Cas3 protein with C-terminal His_6-tag under *araC* based promoter.	Sinkunas et al., 2013[1]
pBAD-Cas8e-C-His	Contains Cas8e protein with C-terminal His_6-tag under *araC* based promoter.	This study
pSP1-AA	Specific target with PAM AA for combined magnetic tweezers and fluorescence measurements at low torque	Sinkunas et al., 2013[1]
pSP3-AA	Non-specific target with PAM AA	Sinkunas et al., 2013[1]
**Bacterial strains**	**Description**	**Reference**
Escherichia coli DH5a	Used for plasmid cloning, plasmid purification	Thermo Fisher Scientific, #18265017
Escherichia coli BL21 (DE3)	Used for protein expression	New England Biolabs, #C2527

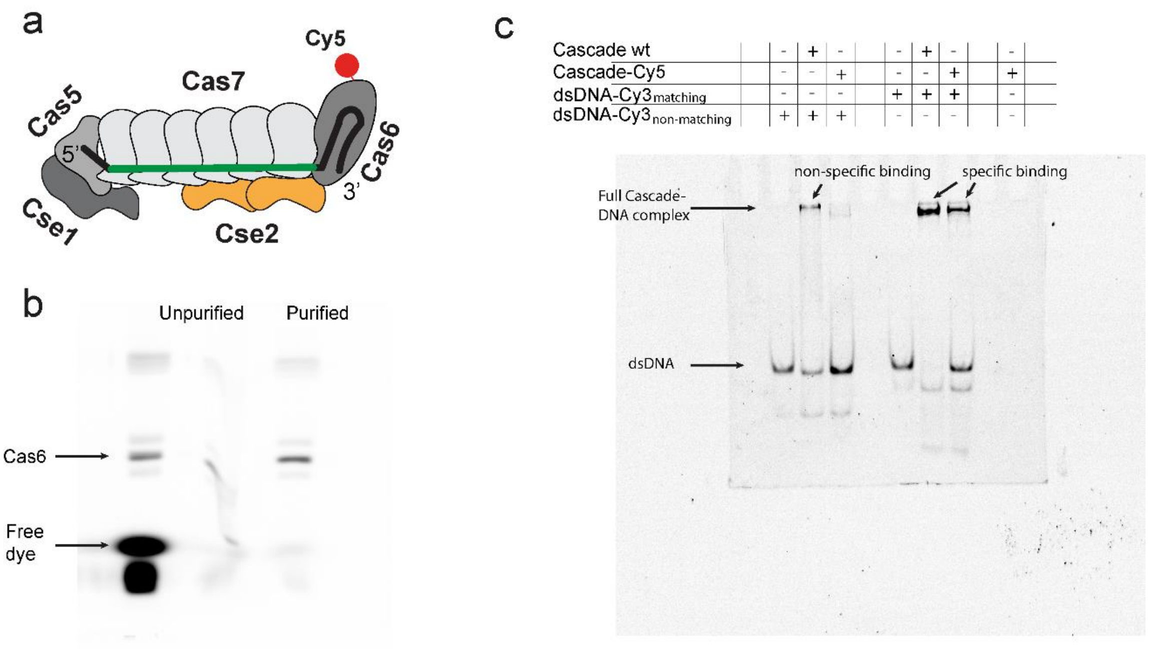

Figure S1. Labeling St-Cascade with a Cy5 dye for TIRF measurements. a Labeled St-Cascade complex. The complex is labeled via a malemeide linker on the Cas6 protein. b SDS-PAGE showing the successfully labeled Cas6 before and after purification. The experiment was performed once. c Electrophoretic Mobility Shift Assay. St-Cascade labeled with Cy5 and wild-type binding behaviour on matching and non-matching dsDNA targets labeled with Cy3. Both St-Cascade variants bind to the matching target. Slight non-specific binding is observed for wt Cascade but not for labeled Cascade (Cascade-Cy5). The image was taken in the green fluorescence channel (~530nm) in order to make the DNA visible. The experiment was repeated three times indepently with similar results.

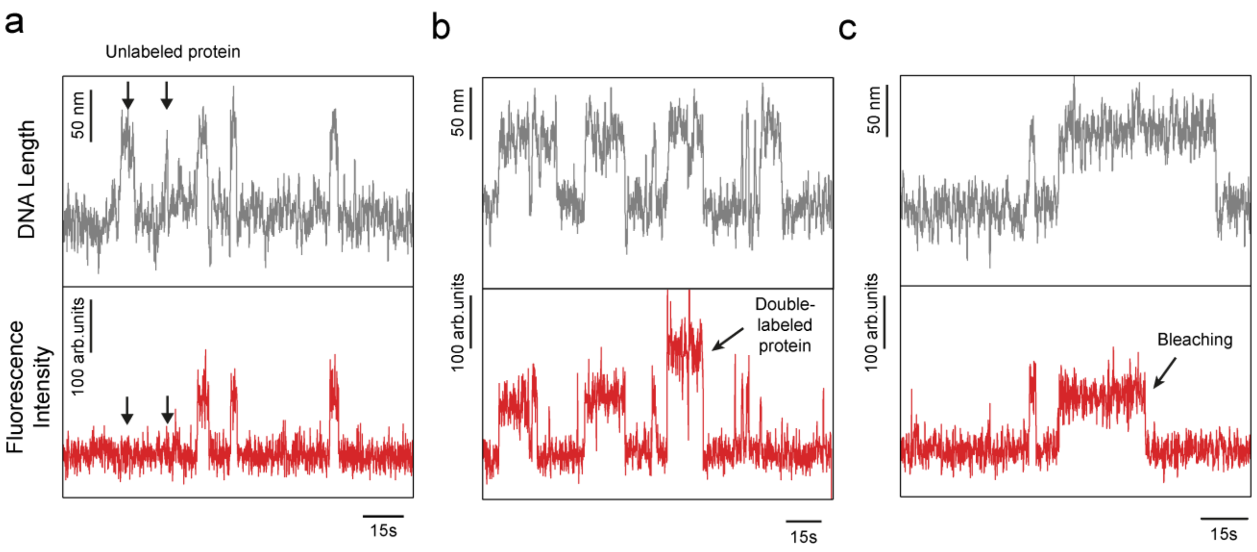

Figure S2. Correlated trajectories magnetic tweezers and fluorescence trajectories monitoring unlabeled and dimeric Cascade complexes as well as fluorophore bleaching. All shown trajectories were taken at 0.2pN and -6 turns (Γ = -4.7 pN nm). The DNA length trajectories (grey) were taken at 120 Hz and smoothed to 3Hz while the fluorescence trajectories (red) were recorded at 10 Hz. **a** R-loop formation events without detectable binding events (indicated by black arrows) indicate the binding of unlabeled St-Cascade complexes. By counting the binding events of labeled and unlabled complexes, a labeling efficiency of St-Cascade of 65±10% was determined from the single-molecule experiments. **b** Occasional observation of binding events (7% of events) with doubled intensity (black arrow) indicated the binding of doubly labeled St-Cascade or a St-Cascade dimer. These events were considered in our quantitative analysis as they did not show an altered behavior **c** Occasional disappearance of the fluorescence signal before R-loop collapse was attributed to bleaching of the Cy5-fluorophore. In the analysis, these events were included for determining the delay between binding and R-loop formation but not for dissociation.

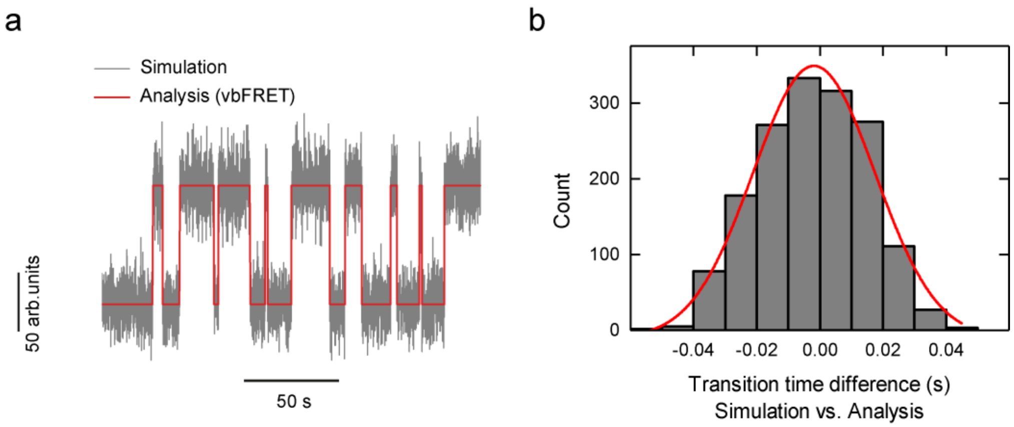

Figure S3 Analysis of transition times in simulated fluorescence trajectories. a Simulated fluorescence trajectories (grey) comprising multiple binding-dissociation events with an average length of 10 s. The two-state approximation of the trajectory from Hidden Markov modelling is shown in red. **b** Distribution of the error of individual transition times determined from the simulated trajectories (grey bars, $N = 1602$). A Gaussian distribution is depicted in red. It provided a mean of 1ms and a standard deviation of 17 ms. Source Data are provided as a Source Data file.

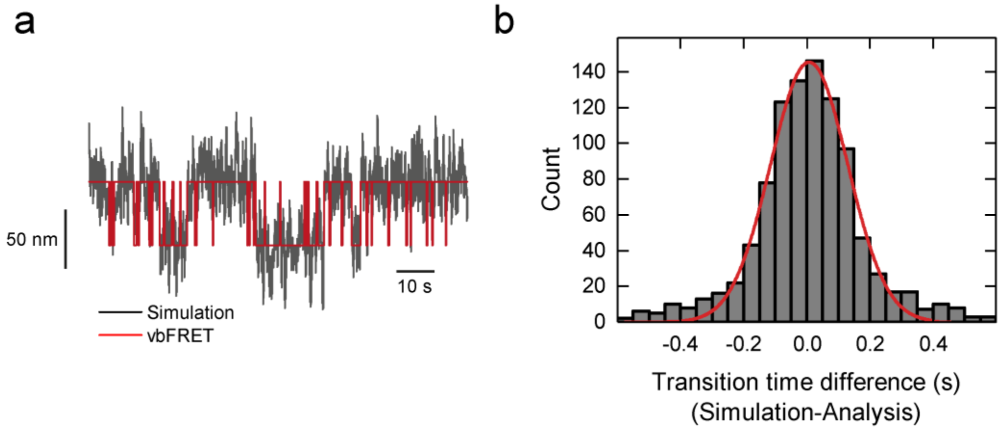

Figure S4 Analysis of transition times in simulated magnetic tweezers trajectories. a Simulated magnetic tweezers trajectory with multiple R-loop formation events (black) and a two state approximation of the trajectory from Hidden Markov modeling (red). **b** Difference between the nominal transition times from the simulated trajectory and the transition times found in the simulated trajectories. The histogram of the values is depicted in black, while the maximum likelihood function is depicted in red. Source Data are provided as a Source Data file.

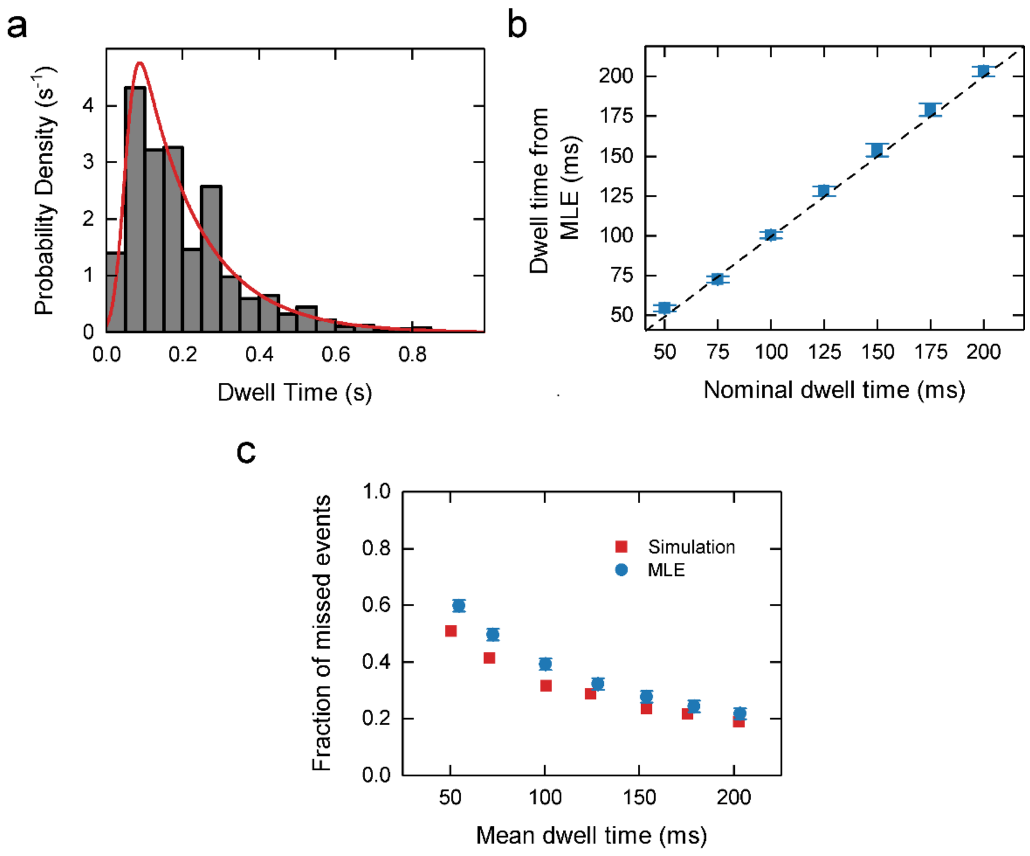

Figure S5. Estimating the fraction of undetected short binding events by MLE. a Dwell time histogram obtained from simulated fluorescence trajectories (50 ms integration time) using a nominal dwell time of 154 ms (grey bars, *N* = 1865). The result of the MLE analysis is shown as a red line providing $\tau = 154 \pm 4$ ms and $\mu = 53 \pm 10$ ms **b** Mean dwell times found by the MLE analysis compared to the nominal mean dwell times used in the simulations (blue squares, *N*=1221, 1444, 1710, 1718, 1865, 1926 and 1952, respectively, from low (50ms) to high (200ms) nominal dwell times). The dotted line corresponds to the identity between both axis values. Errors correspond to SEM obtained from MLE. **c** Fraction of missed events retrieved from the MLE analysis (blue circles) compared to the actual fraction of missed events by Hidden Markov modelling (red squares). The number of individual simulated binding events corresponds to *N*=2484, 2459, 2499, 2407, 2440, 2457 and 2417, respectively, from low (50ms) to high (200ms) nominal dwell times. Errors correspond to SEM obtained from MLE. Source Data are provided as a Source Data file.

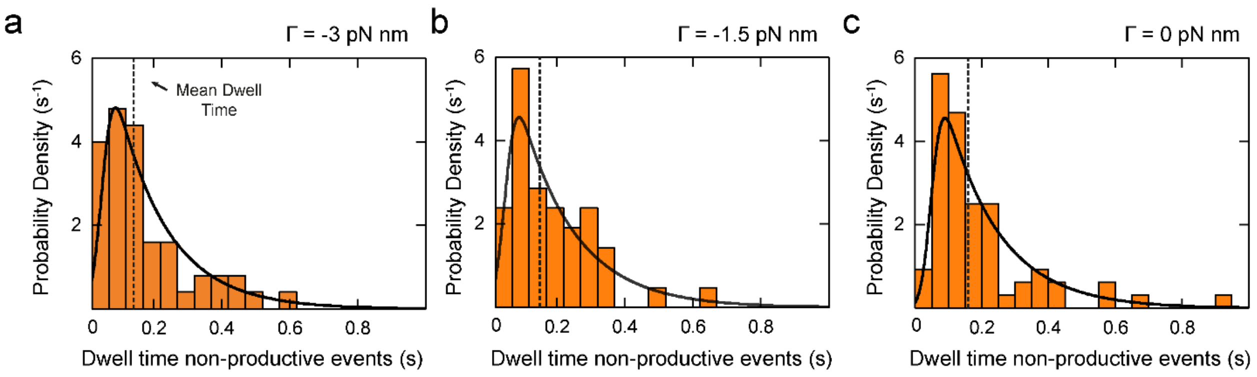

Figure S6. Dwell time histograms of the non-productive binding events for different torques. The black lines represent the maximum likelihood estimate of the data, while the dashed line indicates the mean dwell time. **a** Histogram for Γ = -3 pN nm (N = 50, τ_{mean} = 135 ± 19 ms). **b** Histogram for Γ = -1.5 pN nm (N = 50, τ_{mean} = 145 ± 23 ms). **c** Histogram for Γ = 0 pN nm (N = 114, τ_{mean} = 160 ± 17 ms). Source Data are provided as a Source Data file.

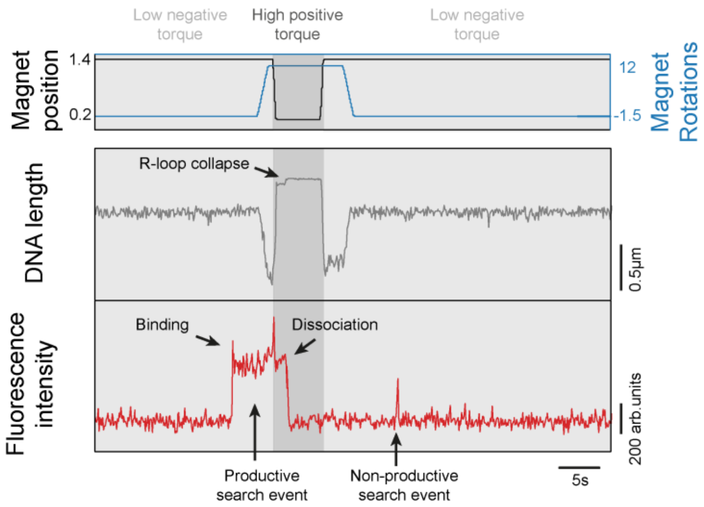

Figure S7. Correlated DNA length and fluorescence trajectories with productive and non-productive search events at low torque (Γ= -1.5 pN nm, F = 0.2 pN). The DNA length (grey) trajectory was recored at 120 Hz and smoothed to 10 Hz, while the fluorescence trajectory (red) was recorded at 20Hz. Long lasting binding events corresponded to productive target search events yielding a locked R-loop. Locked R-loop formation was verified and reversed by transiently applying a high positive torque (dark grey shaded area, Γ= 15 pN nm, F = 1.6 pN). This promoted R-loop collapse seen as a pronounced DNA length jump. In contrast, short binding events corresponded to non-productive search event without R-loop formation.

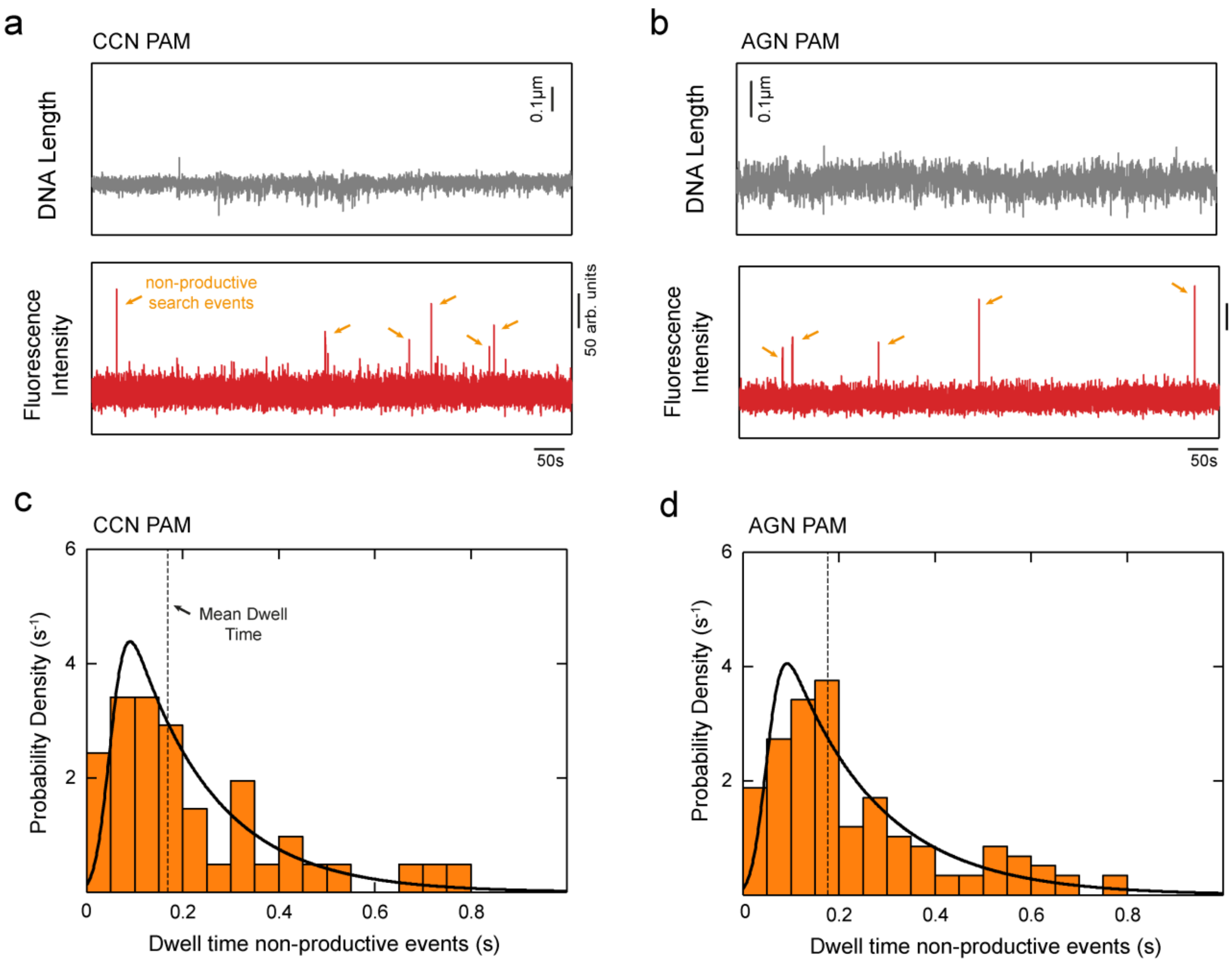

Figure S8. Correlated DNA Length and fluorescence trajectories and dwell time histograms of the non-productive binding events for targets with non-cognate PAMs. **a** Trajectories for the non-cognate CCN PAM recorded at Γ = -4.7 pN nm (F=0.2 pN, -4 turns). The orange arrows point at non-productive search events. The DNA length was recorded at 120 Hz and smoothed to 3 Hz. The fluorescence signal was recorded at 20 Hz. **b** Trajectories for the non-cognate AGN PAM recorded at Γ = -4.7 pN nm(F=0.2 pN, -5 turns). **c** Histogram for a CCN PAM (N =41, τ_{mean} = 170 ± 30 ms). Black lines represent the maximum likelihood estimate of the data, while dashed lines indicate the mean dwell time. The data was taken at a torque of Γ = -4.7 pN nm. **d** Histogram for a AGN PAM (N = 119, τ_{mean} = 180 ± 20 ms). Black lines represent the maximum likelihood estimate of the data, while dashed lines indicate the mean dwell time. The data was taken at a torque of Γ = -4.7 pN nm. Source Data are provided as a Source Data file.

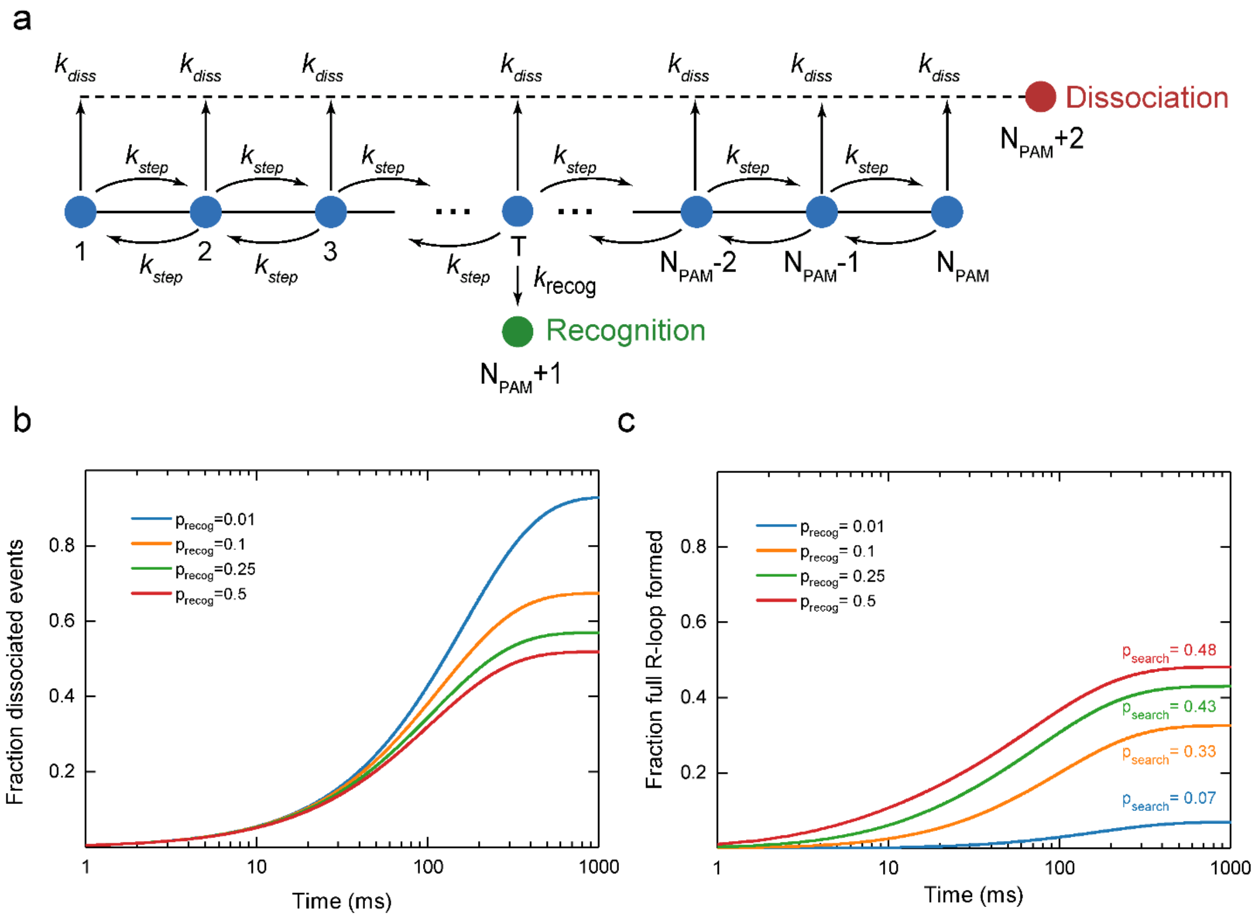

Figure S9 Simulation of the target search model. a The N_{PAM} sequential states represent permissive PAMs with one of the PAMs (T) containing the adjacent target sequence. After random binding to any of the PAMs, Cascade stochastically transits to either of the two next-neighbor PAMs at equal rates k. At every PAM, Cascade can dissociate with rate k_{diss}, corresponding to an irreversible transition to State $N_{PAM}+2$. At the target PAM, T, Cascade can additionally recognize the target with rate k_{recog}, corresponding to an irreversible transition to state $N_{PAM}+1$. **b** Kinetics of non-productive target search events for which Cascade dissociated without recognizing the target. **c** Kinetics of productive target search events for which Cascade recognized the target. The model was solved for a lattice length $N=60$ and different target recognition probabilities per target binding p_{recog}. The fraction of productive events that is asymptotically approached at long times corresponds to the efficiency p_{search} of the target recognition process. Source Data are provided as a Source Data file.

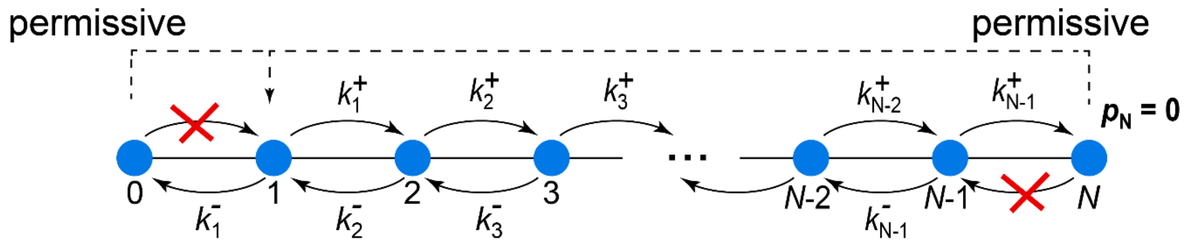

Figure S10 Scheme of the one-dimensional random walk model for target recognition. The probability to reach position N when starting from position 1 without returning to zero is calculated. This problem can be treated by introducing permissive boundaries at position 0 and N. Particles that reach either of these positions are instantaneously placed back to position 1. The probabilities to reach either position N or position 0 are proportional to the fluxes in either direction.

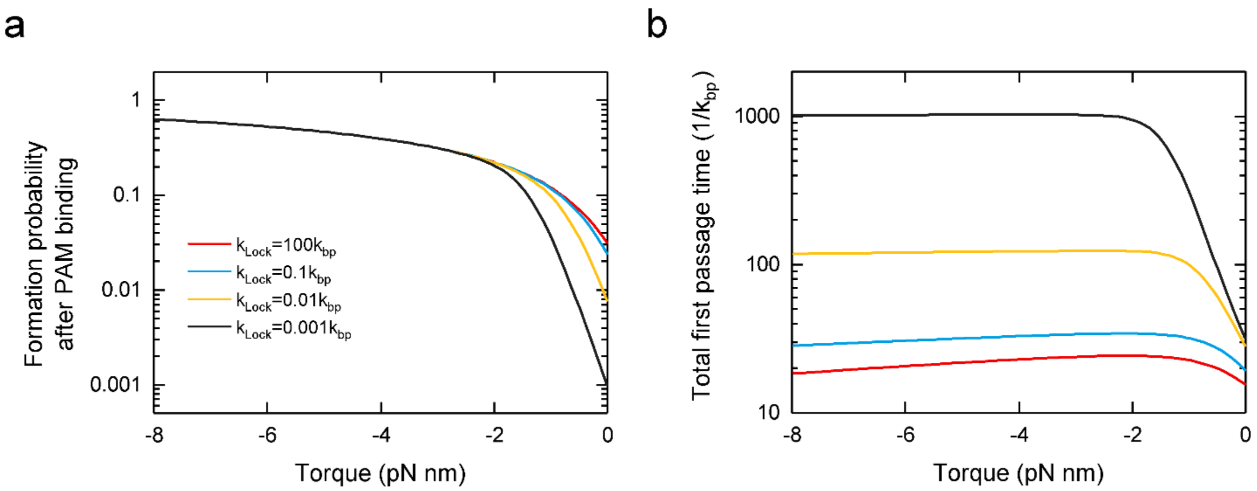

Figure S11 Probability of locked R-loop formation as well as the mean duration of an R-loop formationevent as function of the bias of the energy landscape. **a** Modeled formation probability after PAM binding as function of torque for different locking rates. **b** Total first passage time for R-loop formation function of torque. Shown are the numerical results for an R-loop length N = 32 and various locking rates, where k_{Lock} = 100k_{bp} corresponds to the limit of very fast locking, i.e., negligible time. Source Data are provided as a Source Data file.

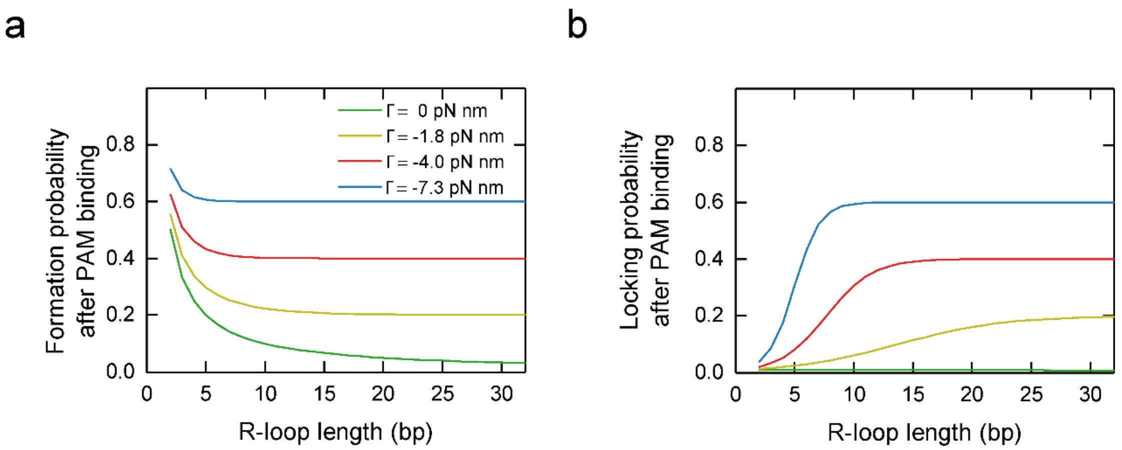

Figure S12 Target recognition probability upon PAM binding as function of the R-loop length. (Left) Formation probability of an unlocked R-loop vs. R-loop length plotted for differently biased energy landscapes. (Right) Probability for locked R-loop formation vs. R-loop length using a locking rate k_{Lock}= 0.01k_{bp}. The biases of the energy landscapes are colored as in the left plot. Source Data are provided as a Source Data file.

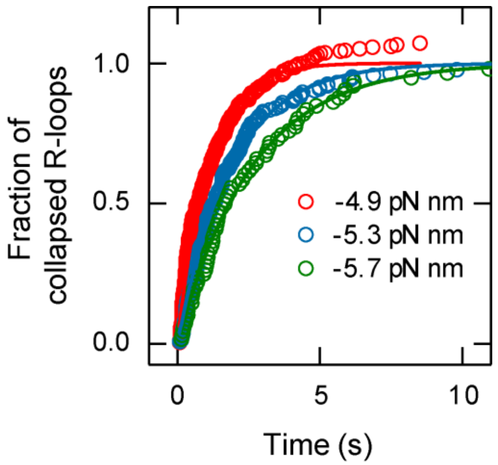

Figure S13 Cummulative probability that a fully formed R-loop has collapsed to the intermediate state as function of time exemplary for two terminal mismatches. Experimental results of individual collapse events for varying torques (-4.9 pN nm, red circles, N= 230; -5.3 pN nm, blue circles, N=160; -5.7 pN nm, green circles, N=68). Single exponential fits are depicted as lines in the respective colours. Source Data are provided as a Source Data file.

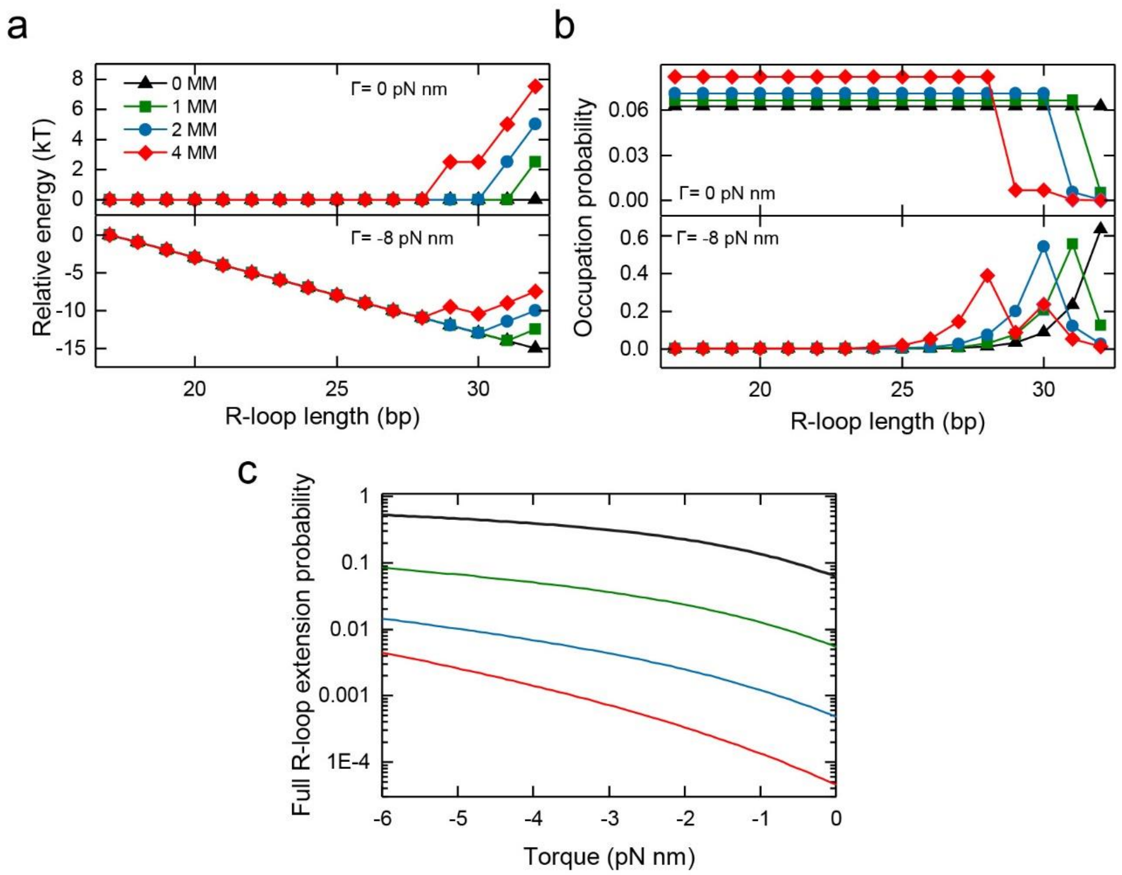

Figure S14 Modeling the energy landscapes of R-loop formation by St-Cascade. a Model energy landscapes of R-loop formation for different numbers of PAM-distal mismatches at zero torque (top) and at a torque of Γ = -8 pN nm using a mismatch penalty of $\Delta G_{MM} = 2.5\ k_b T$. The depicted free energy is relative to the free energy at position 17. **b** Occupation probabilities of the R-loop length calculated for the different energy landscapes in **a** at zero torque and at a torque of Γ = -8 pN nm. **c** Occupation probability of the full R-loop length, i.e. the probability that the R-loop extends to a length of 32 bp, as function of torque for different numbers of PAM-distal mismatches. Source Data are provided as a Source Data file.

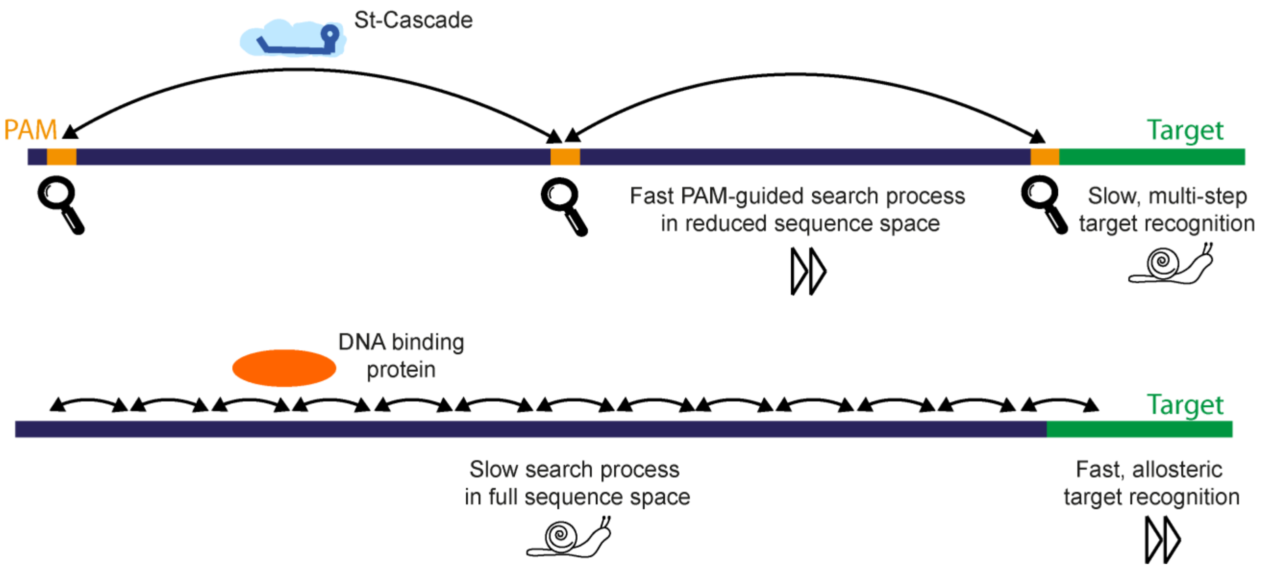

Figure S15. Schematic representation of proposed DNA search by St-Cascade and other DNA binding proteins. The St-Cascade target search is limited to the basepairs directly adjacent to PAMs, allowing to ignore large parts of the DNA sequence space. This accelerated target search potentially makes up for the slow, multi-step target recognition by R-loop formation. In contrast, other DNA binding proteins search the full sequence space for a target, which is then recognized through an allosteric single-step process.

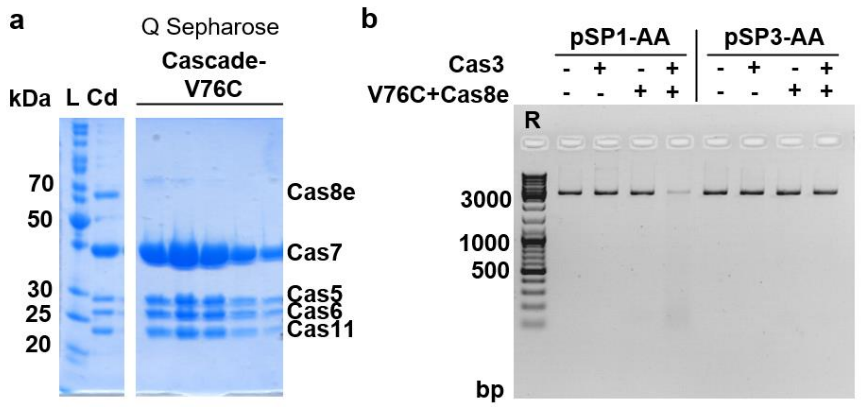

Figure S16. Purification and cleavage assays of Cascade-Cas6-V76C effector complex. a. 12 % SDS-PAGE after purification of Cascade through Q-Sepharose column. L represents PageRuler™ Unstained Protein Ladder, Cd – the wt Cascade effector complex as described in (Sinkunas et al., 2013). Next lines represent a peak of Cascade-Cas8e-C252S-C262S-Cas6-V76C complex (later on called Cascade-Cas6-V76C) on Q Sepharose column. Cas8e C252S-C262S mutant is missing from the complex. b. Cascade-Cas6-V76C activity assay with Cas3 nuclease-helicase. R stands for MassRuler DNA Ladder Mix. 3 nM pSP1-AA (specific target) or pSP3-AA (non-specific target), 100 nM Cas3, 100 nM Cascade and 100 nM Cas8e-C-His (or their buffers, respectively) incubated in the Nuclease buffer (10 mM Tris–HCl (pH 7.5), 75 mM NaCl, 40 mM KCl, 7% (v/v) glycerol, 1.5 mM MgCl2, 0.1 mM NiCl2, 2 mM ATP). The reaction was started by adding Cas3 to the mixture, incubated at 37°C and stopped after 40 min. Reactions were stopped by adding 1/3 of the reaction volume of “stop solution” containing 67.5 mM EDTA, 27% (v/v) glycerol, 0.3% (w/v) SDS and 0.1% w/v Orange G. The reaction products were visualized on 0.8 % agarose DNA gel and a voltage of 3 V/cm followed by visualization using ethidium bromide staining. The experiments were conducted once, i.e. not reproduced. However, binding activity was measured twice more, indepently (see Supplementary Fig. 1).

Supplementary Notes

1 Quantifying the accuracy of the transition point determination in fluorescence and magnetic tweezers trajectories

The determined dwell times connected to the target search process were in the range of the resolution limits of the magnetic tweezers assay and the fluorescence signal acquisition. Extraction of reliable dwell time estimates required an elaborate data analysis procedure. Both the fluorescence and the DNA length trajectories exhibited considerable noise that limited the accuracy at which transition points could be determined. To quantify the accuracy of the transition point determination, we used simulated fluorescence and DNA length trajectories including transitions and performed our data analysis in the same manner as for the experimental data. This allowed us to obtain realistic dwell time estimates as well as errors.

Accuracy of transition points in fluorescence trajectories

The accuracy of transition points in fluorescence trajectories was mainly limited by the Poisson distributed shot noise of the photon statistics[2] as well as the finite camera integration time of 50 ms. To simulate fluorescence trajectories, we first simulated two-state binding/dissociation trajectories obeying first order kinetics using k_{bind}= 0.05 s^{-1} and k_{diss}= 0.1 s^{-1} at a time resolution of 0.5 ms. We then split the trajectories into successive 50 ms windows and drew for each window random photon numbers from a Poisson distribution. The expected (mean) photon number λ depended on the relative length of the unbound and bound states within each window. We assumed average photon numbers of $\lambda = 250$ and $\lambda = 350$ if the time window was fully occupied by the unbound state and the bound state, respectively. This provided a signal change of 100 photons upon binding, such that we obtained signal-to-noise ratios of $SNR \approx \Delta\lambda/\sqrt{\lambda} \approx 10$ as experimentally observed (Supplementary Fig. 3a).

We next analyzed the transition points in the simulated fluorescence trajectories in the same manner as the experimental trajectories using a Hidden Markov modelling algorithm[3]. The time error in determining the transition points was then calculated from the difference between input transition points for the simulations and output transitions from the analysis. The transition time errors were approximately Gaussian distributed with a mean of $\mu_{Fl} = 1.0 \pm 0.5$ ms indicating that on average the positions of transition points were correctly determined. The standard deviation of the distribution, corresponding to the accuracy of a single measured transition time, was $\sigma_{Fl} = 17$ ms (Supplementary Fig. 3b).

Accuracy of transition points in magnetic tweezers trajectories

The accuracy of transition times in DNA length trajectories was mainly limited by the response time of the DNA tethered magnetic bead to sudden DNA length changes as well as thermal DNA length fluctuations. To simulate DNA length trajectories with R-loop formation-collapse events, we first simulated trajectories with sudden (R-loop) transitions between two fixed DNA lengths obeying first order kinetics with $k_{R,form}$= $k_{R,coll}$ = 0.05s^{-1} at a frame rate of 120 Hz. The length difference between the two states was 60 nm as measured for experimentally observed transitions. To simulate DNA length fluctuations, we approximated our nanomechanical system as a sphere being attached to a linear spring with spring constant k that is moving along the z coordinate with drag coefficient γ. The spring constant was determined from the mean-squared fluctuations of the DNA length in experimental trajectories of supercoiled DNA as $k = k_B T/\langle z^2 \rangle = 7.1 \cdot 10^{-4} \mathrm{pN\,nm^{-1}}$. The drag coefficient was then determined from the measured characteristic cut-off frequency $f_c = 12\,Hz$ of the frequency spectrum of the length fluctuations[4] as $\gamma = k/2\pi f_c = 9.4 \cdot 10^{-6}\ \mathrm{pN\,nm^{-1}\,s}$. DNA length fluctuations including R-loop transitions were then modelled using Brownian dynamics simulations[4] in which the two-state R-loop trajectories provided the equilibrium position for the spring extension. (Supplementary Fig. 4a).

We next analyzed the transition times in the simulated trajectories using Hidden Markov modelling[3] and calculated their errors with respect to the nominal transition times. The transition time errors were Gaussian distributed with a pronounced non-zero mean of $\mu_{MT} =$ 13 ms and a standard deviation of σ_{MT} =137 ms (Supplementary Fig. 4b). The measured time delay was due to the finite response time $\tau = \gamma/k =$ 13.2 ms which the nanomechanical system required to adapt to changes of the DNA length. For the collapse of the R-loop, a similar error distribution was obtained. In the DNA length trajectories, the vbFRET algorithm detected more transitions than were present. Excess transitions were removed manually, similar as for the experimental trajectories, for which the fluorescence signal provided a corrective.

2 Maximum Likelihood Estimation of short dwell times

The determined mean values of the dwell times $\tau_{R,form}$, $\tau_{R,coll}$ and $\tau_{non-prod}$ were smaller or on the order of the width of the experimentally determined dwell time distributions. To obtain accurate estimates of the mean dwell times we applied maximum likelihood estimation (MLE). Assuming that the

target search follows first order kinetics, the actual dwell times are exponentially distributed around the mean dwell time τ, which corresponds to the decay time of the exponential:

$$p_\tau(t) = \frac{1}{\tau}\exp\left(-\frac{t}{\tau}\right) \tag{S1}$$

Each experimentally determined dwell time was calculated from the difference of two transition times with their own Gaussian distributed statistical errors as determined above. The experimentally determined dwell times are then, according to the central limit theorem, Gaussian distributed around the actual values, whose mean and variance equal the sum of the means and variances of the individual error distributions, respectively. Correspondingly, the dwell time error distribution of the non-productive events had a mean of $\mu = 2\mu_{Fl} \approx 0\ ms$ and a variance of $\sigma^2 = 2\sigma_{Fl}^2 = (24\ ms)^2$ since it was calculated from two transitions in the fluorescence trajectories. The dwell time error distributions for the productive events had a mean of $\mu = \mu_{Fl} + \mu_{MT} \approx 13\ ms$ and a variance of $\sigma^2 = \sigma_{Fl}^2 + \sigma_{MT}^2 = (138\ ms)^2$. In base cases the dwell time error distributions are described by:

$$p_{error} = \frac{1}{\sigma\sqrt{2\pi}}\exp\left(-\frac{(t-\mu)^2}{2\sigma^2}\right) \tag{S2}$$

The error distributions shift and broaden the actual exponential dwell time distribution p_τ as observed in the experiments. The expected measured dwell time distribution $p_{\tau,exp}$ can be mathematically described as a convolution of p_τ with the corresponding error distribution p_{error}:

$$p_{\tau,meas} = p_\tau * p_{error} \tag{S3}$$

Inserting the distributions and solving the convolution integral provides:

$$\begin{aligned} p_{\tau,exp}(t) &= \int_0^\infty \frac{1}{\tau\sigma\sqrt{2\pi}}\exp\left(-\frac{(t-\mu-t')^2}{2\sigma^2}\right)\exp\left(-\frac{t'}{\tau}\right)dt' \\ &= \frac{1}{2\tau}e^{\frac{1}{2\tau}\left(2\mu+\frac{\sigma^2}{\tau}-2t\right)}\,\mathrm{erfc}\left(\frac{\mu+\frac{\sigma^2}{\tau}-t}{\sqrt{2}\sigma}\right), \end{aligned} \tag{S4}$$

where τ is the mean of the exponential distribution and μ and σ are the mean and standard deviation of the of the Gaussian distributed errors, respectively. The probability of measuring a set of n experimental dwell times t_i is described by the likelihood function

$$L = \prod_i p_\tau(t_i) = \prod_i \frac{1}{2\tau}e^{\frac{1}{2\tau}\left(2\mu+\frac{\sigma^2}{\tau}-2t_i\right)}\,\mathrm{erfc}\left(\frac{\mu+\frac{\sigma^2}{\tau}-t_i}{\sqrt{2}\sigma}\right). \tag{S5}$$

Taking the logarithm of the likelihood function simplifies the product and yields a sum:

$$\ln(L) = \sum_i \ln\left[\frac{1}{2\tau} e^{\frac{1}{2\tau}\left(2\mu + \frac{\sigma^2}{\tau} - 2t_i\right)} \text{erfc}\left(\frac{\mu + \frac{\sigma^2}{\tau} - t_i}{\sqrt{2}\sigma}\right)\right]. \quad \text{(S6)}$$

To find the most likely mean dwell time τ describing the measured dwell time distribution, $\ln(L)$ was numerically maximized for the particular set of data points t_i and error parameters μ and σ. The estimated standard errors of μ and σ were derived from the inverted Hessian matrix of the optimized function, i.e. the covariance matrix[5].

3 Estimating the number of short non-detected binding events of St-Cascade to DNA

The average dwell times for non-productive binding of St-Cascade to DNA were on the order of 150 ms. Given a frame rate of 20 Hz in the measurements, a fraction of short-lived events remained undetected. To reliably determine the search efficiency, we used our MLE approach to estimate the number of undetected events. Assuming that only events with a dwell time larger than a cut-off t_c are detected, the dwell time distribution of the detected events corresponds to an exponential distribution that is shifted by t_c to larger times. Convolution with the error distribution would smoothen the sharp transition around t_c and yield reduced event numbers at low times (Supplementary Fig. 5a), as also seen experimentally (Fig. 2e, main text). To obtain a nominal dwell time distribution, the shift by t_c can be achieved by setting the mean of the Gaussian error distribution to $\mu = t_c$. It can be shown that the fraction of non-detected events among all binding events is then given by:

$$r_{missed} = 1 - e^{\frac{t_c}{\tau}}. \quad \text{(S7)}$$

where τ is the nominal mean dwell time. Using τ and t_c $(\text{i.e.}\,\mu)$ as free parameters in our MLE approximation of the experimental dwell times allowed us to estimate t_c and thus to correct for the fraction of non-detected non-productive binding events.

To validate this approach, we simulated fluorescence trajectories with short binding events using exponentially distributed dwell times of mean τ (see above as described above). Detection of the binding events in the simulated trajectories and analysis of the dwell times (see above) provided dwell time distributions with a significantly reduced number of events at low dwell times, which could be well approximated by the expected dwell time distribution with detection cutoff t_c (Supplementary Fig. 5a). We carried out the simulations and the analysis for a range of different dwell times and verified that the analysis returned, within error, the input dwell times (Supplementary Fig. 5b). Most importantly, from the simulations we could also determine the actual number of events that were missed by the Hidden-Markov

analysis of the trajectories and compare it with MLE result. This revealed that the MLE provided realistic fractions of missed events over the whole range of tested dwell times albeit with a slight overestimation (Supplementary Fig. 5c).

4 Target search model

Following the binding to a random PAM on the DNA, we modeled the target search by St-Cascade as a one-dimensional random walk with stepping rate k_{step} from one PAM to the next adjacent PAM in either direction (Supplementary Fig. 9a). The 1D PAM lattice was limited to N_{PAM} lattice points to mimic the limited illuminated DNA length. The boundaries in our model are considered reflective. One of the PAMs (the target PAM, position 20, taken from the actual DNA sequence used in the experiments) was selected to contain the adjacent target. The mean-squared-displacement of such a discrete random walk is provided by:

$$\langle x^2 \rangle = 2k_{step}\Delta^2 t = 2Dt \qquad \text{(S8)}$$

with $\Delta =$ 2.7 nm being the mean distance between the PAMs. $\langle x^2 \rangle$ has to match the observed mean-square displacement, such that the stepping rate could be obtained from the experimentally determined diffusion coefficient as $k_{step} = \frac{D}{\Delta^2} = 1920\ s^{-1}$. It shall be noted that Equation S8 is valid for unbounded motion only. The eqution is used to infer the the stepping rate of St-Cascade from the lateral diffusion measurements. In the experiment, a DNA substrate of approximately 5µm (15kbp) length was used. Given the finding that St-Cascade scans on average 90nm per 1D search, this assumption is believed to be valid.

At each PAM, St-Cascade could furthermore dissociate from the DNA with dissociation rate k_{diss} =5.7 s^{-1} (Supplementary Fig. 9 and main text) yielding a non-productive search event. Upon arrival at the target PAM at position T, St-Cascade could form an R-loop at rate k_{recog}, yielding a productive event, which is related to the target recognition probability as $p_{recog} = \frac{k_{recog}}{k_{recog}+k_{diss}+2k_{step}}$.

Our model system can be seen as a Markov process with $N_{PAM}+2$ states, where state $N_{PAM}+1$ represents the productive R-loop state and state $N_{PAM}+2$ the unproductive dissociated state. Let $\vec{p}(t)$ be the vector containing the occupation probabilities of all states at any time t, the kinetics of $\vec{p}(t)$ and thus the search process is then described by[6]:

$$\frac{d}{dt}\vec{p}(t) = \overline{\overline{K}}\,\vec{p}(t)\ , \qquad \text{(S9)}$$

where $\overline{\overline{K}}$ is the rate matrix containing the rate constants $k_{i,j}$ of all possible transitions between all states i, j of the system. For the scheme in Figure S9a, the rate matrix has the following form:

$$\begin{array}{c} \\ \overline{\overline{K}} = \end{array} \begin{array}{c} \begin{array}{cccccccc} 1 & 2 & \dots & \mathrm{T} & \dots & N_{PAM} & & \end{array} \\ \begin{pmatrix} -k_{step}-k_{diss} & k_{step} & \dots & 0 & \dots & 0 & 0 & 0 \\ k_{step} & -2k_{step}-k_{diss} & \dots & 0 & \dots & 0 & 0 & 0 \\ 0 & k_{step} & \dots & 0 & \dots & 0 & 0 & 0 \\ \dots & \dots & \dots & \dots & \dots & \dots & \dots & \dots \\ 0 & 0 & \dots & k_{step} & \dots & 0 & 0 & 0 \\ 0 & 0 & \dots & -2k_{step}-k_{diss}-k_{recog} & \dots & 0 & 0 & 0 \\ 0 & 0 & \dots & k_{step} & \dots & 0 & 0 & 0 \\ \dots & \dots & \dots & \dots & \dots & \dots & \dots & \dots \\ 0 & 0 & \dots & 0 & \dots & k_{step} & 0 & 0 \\ 0 & 0 & \dots & 0 & \dots & -k_{step}-k_{diss} & 0 & 0 \\ 0 & 0 & \dots & k_{recog} & \dots & 0 & 0 & 0 \\ k_{diss} & k_{diss} & \dots & k_{diss} & \dots & k_{diss} & 0 & 0 \end{pmatrix} \end{array} \begin{array}{c} 1 \\ 2 \\ 3 \\ \dots \\ T-1 \\ T \\ T+1 \\ \dots \\ N_{PAM}-1 \\ N_{PAM} \\ N_{PAM}+1 \\ N_{PAM}+2 \end{array} \qquad \text{(S10)}$$

Eq. S9 is solved by

$$\vec{p}(t) = \exp\left(\overline{\overline{K}}t\right)\vec{p}(0) \qquad \text{(S11)}$$

with $\exp\left(\overline{\overline{K}}t\right)$ being the matrix exponential of $\overline{\overline{K}}$ and $\vec{p}(0)$ being the initial probability distribution at $t = 0$. The exponential term can be expressed as

$$\exp\left(\overline{\overline{K}}t\right) = \bar{\bar{x}} \cdot \exp\left(\overline{\overline{\Lambda}}t\right) \cdot \bar{\bar{x}}^{-1} \;, \qquad \text{(S12)}$$

where $\bar{\bar{x}}$ is the eigenvector matrix of $\overline{\overline{K}}$, and $\exp\left(\overline{\overline{\Lambda}}t\right)$ is a diagonal matrix, containing exponential decays in time with the eigenvalues λ_i as decay constants:

$$\exp\left(\overline{\overline{\Lambda}}t\right) = \begin{pmatrix} \exp(\lambda_1 t) & 0 & \dots \\ 0 & \exp(\lambda_2 t) & \dots \\ \dots & \dots & \dots \end{pmatrix}. \qquad \text{(S13)}$$

The entries of the matrix exponential are, according to Eq. S12, sums of the $N + 2$ exponential decays:

$$\left(\bar{\bar{x}} \cdot \exp\left(\overline{\overline{\Lambda}}t\right) \cdot \bar{\bar{x}}^{-1}\right)_{i,j} = \sum_{k=1}^{N_{PAM}+2} x_{i,k} x_{k,j}^{-1} \exp(-\lambda_k t). \qquad \text{(S14)}$$

Combining Eqns. S11, S12 and S14, provides then for the occupation probability of state i the following sum of the N_{PAM}+2 exponential decays:

$$p_i(t) = \sum_{k=1}^{N_{PAM}+2} \exp(-\lambda_k t) \sum_{j=1}^{N_{PAM}+2} p_j(0)\, A_{ijk} \qquad \text{(S15)}$$

with $A_{ijk} = x_{i,k}x_{k,j}^{-1}$. For an initial binding of Cascade to a random PAM, the starting probabilities are given by:

$$\vec{p}(0) = (1/N_{PAM}, 1/N_{PAM}, \dots, 1/N_{PAM}, 0,0) \quad \text{(S16)}$$

With this solution and experimentally determined values for k_{step} and k_{diss}, one can calculate the kinetics of productive ($p_{N+1}(t)$) and non-productive ($p_{N+2}(t)$) target recognition events for any set the lattice length N_{PAM} and the target recognition efficiency p_{recog} (Supplementary Fig. 9a). We validated the obtained kinetics using stochastic simulations of the random walk process. The fraction of productive events that is asymptotically approached at long times provides the efficiency p_{search} of the target recognition process (Supplementary Fig. 9c). $p_{search}$was increasing with increasing p_{recog} and generally larger than p_{recog} due to multiple target site revisits during the search. From the kinetics, we furthermore calculated the mean event durations, i.e. the dwell times for non-productive and productive search events:

$$\tau_{R,form/non-prod} = \langle t_i \rangle = \sum_{k=1}^{N_{PAM}+2} 1/\lambda_k \sum_{j=1}^{N_{PAM}+2} p_j(0)\, A_{ijk} \quad \text{(S17)}$$

with i being either $N_{PAM} + 1$ or $N_{PAM} + 2$, respectively.

Dwell times for R-loop collapse were obtained from the calculated kinetics of non-productive events that started at the target site at position T for which the initial occupancies changed to $\vec{p}(0) = (0, \dots, 0,1,0, \dots, 0)$. The number of target site revisits for productive events was obtained from 10,000 stochastic simulations of the target search process following the scheme in Supplementary Fig. 9a. The search process was always started at the target site and the number of revisits was counted for each productive search event.

5 Target recognition model

Probability to finish a 1D random walk once the first step was taken

Due to the reversibility of the R-loop formation process, an R-loop of a CRISPR-Cas effector complex that became nucleated upon PAM binding has, even on a perfectly matching sequence, a certain chance to collapse without full R-loop formation or locking. To provide a theoretical description for the probability p_{recog} at which a matching target becomes recognized upon PAM binding, we model R-loop formation as a one-dimensional random walk process over $N+1$ positions (0,1,2,...,N), where N corresponds to the number of base pairs of the full R-loop (Supplementary Fig. 10). Within the model, position 0 corresponds to the unbound state, position 1 to the PAM-bound state with the first base-pair of the R-loop being formed and the positions from 2 to N to all subsequent R-loop lengths. To include R-loop locking, an additional position $N+1$ can be introduced. Transitions are only allowed between neighboring states. They are described by position-dependent rate constants k_i^+ for forward steps and k_i^- for backward steps with i indicating the position. Within the random walk model, the target recognition following PAM binding translates into a start of the walk at position 1. The walk is terminated when either Position 0 (non-successful recognition) or position N (successful recognition) are reached. The target recognition probability is then the probability p_{pass} for a successful termination of the walk. To obtain this probability but also the time scale of the walk in the frame work of the model, one introduces permissive boundaries at positions 0 and N and places a single particle into the system.

Once the particle reaches a boundary, the particle is instantaneously placed to the start position 1. To obtain average quantities over many single-particle trajectories, we assume steady state conditions for this system, i.e. probabilities p_i for the particle to be found at position i that are time-invariant. Given the permissive boundaries we directly get $p_0 = p_N = 0$. In steady state, this results in a constant backward flux j_- of particles from position 1 to 0 and a constant forward flux j_+ of particles from position 1 to N. The fluxes correspond to the rates of particles arriving at either boundary, i.e. to the rates of successful vs. unsuccessful recognition. The probability p_{pass} to reach position N is then provided by the forward flux divided by the total flux at position 1:

$$p_{pass} = \frac{j_+}{j_+ + j_-} = \frac{1}{1 + \frac{j_+}{j_-}}. \qquad \text{(S18)}$$

For the forward/backward flux $j_i^\pm$ between two neighboring positions i and $i+1$ we can write:

$$j_i^\pm = \pm k_i^+ p_i \mp k_{i+1}^- p_{i+1} \qquad \text{(S19)}$$

In steady state, the probability at any position i does not change in time such that for a pure linear chain we have:

$$\frac{dp_i}{dt} = j_{i+1} - j_i = 0 \qquad \text{(S20)}$$

Recursively, it follows that $j_i = j_0$ for all i of a linear chain segment not containing branches. For the backward flux j_-, we then get from $p_0 = 0$ and Eq. S19:

$$\frac{p_1}{j_-} = \frac{1}{k_1^-} \qquad \text{(S21)}$$

For the forward flux, we get from Eq. S19:

$$\frac{p_i}{j_+} = \frac{1}{k_i^+} + \frac{k_{i+1}^-}{k_i^+}\frac{p_{i+1}}{j_+} \qquad \text{(S22)}$$

Thus, with the boundary condition $p_N = 0$, we can obtain all p_i/j_+ from N-1 to 1 in a recursive manner:

$$\frac{p_{N-1}}{j_+} = \frac{1}{k_{N-1}^+} \ ,$$

$$\frac{p_{N-2}}{j_+} = \frac{1}{k_{N-2}^+} + \frac{1}{k_{N-2}^+}\frac{k_{N-1}^-}{k_{N-1}^+} \ ,$$

$$\frac{p_{N-3}}{j_+} = \frac{1}{k_{N-3}^+} + \frac{1}{k_{N-3}^+}\frac{k_{N-2}^-}{k_{N-2}^+} + \frac{k_{N-2}^-}{k_{N-2}^+}\frac{k_{N-1}^-}{k_{N-1}^+}, \dots$$

Since the probability distribution $\{p_i\}$ is normalized (single particle in the system) and $p_0 = 0$, one can easily derive an expression for the forward flux:

$$\sum_{i=1}^{N}\frac{p_i}{j_+} = \frac{1}{j_+}\sum_{i=0}^{N} p_i = \frac{1}{j_+} \qquad \text{(S23)}$$

The local probabilities are then obtained by multiplying the derived values p_i/j_+ with j_+. From p_1 and Equation S21, one obtains $j_- = k_1^- p_1$. Using the principle of detailed balance, one can express the ratio of the rate constants between positions i and $i+1$ directly as

$$k_{i+1}^-/k_i^+ = \exp(\Delta G_i/k_B T). \qquad \text{(S24)}$$

The variable $\Delta G_i = G_{i+1} - G_i$ is the local bias of the energy landscape, i.e. the free energy difference between positions $i+1$ and i. With this, Eq. S22 becomes:

$$\frac{p_i}{j_+} = \frac{1}{k_i^+} + \frac{p_{i+1}}{j_+}e^{\Delta G_i/k_B T}. \qquad \text{(S25)}$$

In a recursive manner we can obtain all p_i/j_+ as above, for which we obtain the following form final form:

$$\frac{p_{N-i}}{j_+} = \sum_{j=1}^{i} \frac{1}{k_{N-j}^{+}} \, e^{(G_{N-j}-G_{N-i})/k_BT} \quad . \tag{S26}$$

Using this expression, we obtain p_1/j_+ and obtain after transformation:

$$j_+ = \frac{p_1}{\sum_{i=1}^{N-1} \frac{1}{k_{N-i}^{+}} \exp(G_{N-i} - G_1)/k_BT} = \frac{p_1}{\sum_{i=1}^{N-1} \frac{1}{k_i^{+}} \exp(G_i - G_1)/k_BT} \quad . \tag{S27}$$

With $j_- = k_1^- p_1$, we get for the success probability of the random walk:

$$p_{pass} = \frac{1}{1 + k_1^- \sum_{i=1}^{N-1} \frac{1}{k_i^+} \exp \frac{G_i - G_1}{k_BT}} . \tag{S28}$$

Formation probability of a matching R-loop of N base pairs under constant bias

To derive an expression for the formation probability of an (unlocked) R-loop of length N following PAM binding, we assume a constant bias for the energy landscape for all R-loop states such that $G_{i+1} - G_i = E_{bias}$ for $1 \leq i < N$. The energy difference between position i and 1 is given as $G_i - G_1 = (i-1)E_{bias}$. Furthermore, we assume position-independent values for the forward and backward stepping rates. Simplifying the exponential terms of the bias energy in Eq. S28 with the rate ratio between the backward and the forward stepping rate

$$s = \frac{k^-}{k^+} = e^{E_{bias}/k_BT} \tag{S29}$$

yields for the R-loop formation probability:

$$p_{Rloop} = \frac{1}{1 + e^{E_{bias}/k_BT} \sum_{i=1}^{N-1} e^{(i-1)\cdot E_{bias}/k_BT}} = \frac{1}{1 + \sum_{i=1}^{N-1} s^i} \tag{S30}$$

Using the sum formula of the geometric series, one gets

$$p_{Rloop} = \frac{1}{1 + s\frac{s^{N-1} - 1}{s - 1}} = \frac{s - 1}{s^N - 1} \tag{S31}$$

Locked R-loop formation under constant bias

We further consider 'locking' to derive a more realistic target recognition probability that includes the effects of the conformational changes during locking, which are slow compared to the single base pair steps. To this end, we introduce an additional locking step from position N to $N+1$ with the rate constant $k_N^+ = k_L$. The target recognition probability then becomes

$$p_{recog} = \frac{1}{1 + k^- \sum_{i=1}^{N} \frac{1}{k_i^+} e^{(i-1)\, E_{bias}/k_B T}} = \frac{1}{1 + \sum_{i=1}^{N-1} s^i + \frac{k^+}{k_L} s^N} \quad \text{(S32)}$$

This can be further transformed to:

$$p_{recog} = \frac{1}{1 + s \cdot \frac{s^{N-1} - 1}{s - 1} + \frac{k^+}{k_L} s^N} = \frac{s - 1}{s^N \left[1 + (s-1)\left(\frac{k^+}{k_L}\right)\right] - 1} \quad \text{(S33)}$$

Assuming that the activation barriers for single base pair steps of R-loop formation are centered between subsequent R-loop positions, the bias-dependent forward and the backward stepping rates for R-loop formation can be expressed using Arrhenius-like terms:

$$k^{\pm} = k_{bp}\, e^{\mp\frac{E_{bias}}{2}} = k_{bp}\, s^{\mp\frac{1}{2}} \quad \text{(S34)}$$

with k_{bp} being the forward and backward stepping rate in absence of bias. When plotting the target recognition probability as function of the (negative) bias of the energy landscape as applied by the supercoiling, one sees that it strongly increases with the applied bias, similar to the first passage time of R-loop formation (Supplementary Fig. 11). Reducing the locking rate reduces the target recognition probability only for weak but not for strong biases (Supplementary Fig. 11a). This can be better understood by looking at the probabilities of (unlocked) R-loop formation and locked R-loop formation as function of the R-loop length (Supplementary Fig. 12). For an unlocked R-loop formation we have high probabilities to form short R-loops due to the limited length of the random walk which drop with increasing R-loop lengths (Supplementary Fig. 12a). It is important to note that even for a mild downhill bias, the R-loop formation probability saturates at a minimum value already at short R-loop lengths. This means that the decision for formation or collapse of an R-loop is taken at the beginning of the random walk. Once the R-loop has been further expanded, it will for the given bias practically always form but not collapse anymore. This changes somewhat if a considerably slower locking step is added. Locked R-loop formation is particularly disfavored at short lengths, since the slow step represents a high kinetic (activation) barrier that needs to be overcome. The probability then increases with length in presence of a negative bias, reaching the same saturation values as for unlocked R-loops (Supplementary Fig. 12b). Due to the negative bias, the unlocked R-loops are already sufficiently stabilized compared to the activation barrier for locking. Locking is then just an additional step after R-loop formation and occurs without an additional collapse. By contrast, at zero or mild bias, the locking barrier still represents an additional hurdle that can promote R-loop collapse such that the locked R-loop formation is less likely than unlocked R-loop formation.

6 Determination of the apparent locking rate from magnetic tweezers trajectories

In the following we derive a simple formula to calculate the apparent locking rate from the magnetic tweezers trajectory in Fig. 5, main text. When the R-loop is in the full state F, R-loop collapse to the intermediate state I and locking state L are competing:

$$I \xleftarrow{k_I} F \xrightarrow{k_{lock,app}} L \tag{S35}$$

This kinetic scheme can be described by the following set of differential equations:

$$\begin{aligned} \frac{dp_F}{dt} &= -k_I p_F - k_L p_F \\ \frac{dp_I}{dt} &= k_I p_F \\ \frac{dp_L}{dt} &= k_L p_F , \end{aligned} \tag{S36}$$

where p_F, p_I and p_L are the probabilities that the R-loop is in the full, intermediate and locked state respectively. At $t = 0$, i.e. when the full state has just formed, we have the following starting conditions:

$$\begin{aligned} p_F(0) &= 1 \\ p_I(0) = p_L(0) &= 0 \end{aligned} \tag{S37}$$

The first differential equation is a simple first order decay, such that we get with the starting conditions:

$$p_F(t) = e^{-(k_I+k_L)t} \tag{S38}$$

Inserting into the second equation gives:

$$\frac{dp_I}{dt} = k_I e^{-(k_I+k_L)t} \tag{S39}$$

Integration gives for the time-dependent probability p_I that a full R-loop has collapsed to the intermediate state:

$$p_I(t) = p_I(0) + \int_0^t k_I e^{-(k_I+k_L)t} dt = \frac{k_I}{k_I + k_L}\left(1 - e^{-(k_I+k_L)t}\right) \tag{S40}$$

as the only measurable probability. The probability $p_I(t)$ rises only to the amplitude:

$$\underbrace{\frac{k_I}{k_I + k_L}}_{p_{coll}} = 1 - \underbrace{\frac{k_L}{k_I + k_L}}_{p_{lock}} \tag{S42}$$

With p_{coll} or p_{lock} being the probabilities that a formed full R-loop collapses or becomes locked, respectively. The collapse occurs with the rate constant

$$k_{coll} = k_I + k_L \tag{S43}$$

If one determines p_{lock} from the fraction of events that successfully get locked and k_{coll} from fitting the probability of collapse for fully formed R-loop (Supplementary Fig. 13) using Eq. S40, one can calculate k_I and k_L from:

$$\begin{aligned} k_I &= (1 - p_{lock})k_{coll} \\ k_L &= p_{lock}\, k_{coll} \end{aligned} \tag{S44}$$

7 Estimation of the 'real' locking rate

Due to their reversibility, unlocked R-loops do not possess a fixed length but rather sample different lengths dependent on the energy landscape of R-loop formation. The results of our experiments suggested (see main text) that R-loop locking is only triggered if the R-loop extends over its full length. Given that the unlocked R-loop samples the full length only for a fraction of the time, the measured torque-dependent apparent locking rate would be considerably lower than the real locking rate k_{lock}, i.e., the rate at which the locking transition occurs, once a full R-loop has been formed. The relation between both rates would be given as:

$$k_{lock,app} = p_{32}\, k_{lock}\ , \tag{S45}$$

where p_{32} is the probability that the R-loop extends all the way to the last base pair. The probabilities p_i that an R-loop extends over i base pairs can be determined from the energy landscape of R-loop formation using the Boltzmann distribution:

$$p_i = \frac{1}{Z} e^{-G_i/k_B T} \qquad \text{with} \qquad Z = \sum_{i=1}^{32} e^{-G_i/k_B T} \tag{S46}$$

being the partition function Z that ensures normalization of the probabilities for all states. As described above, we assumed an energy landscape with a constant negative bias from the applied supercoiling. For each PAM-distal mismatch, a (positive) free energy penalty was introduced at its position[7,8] since disrupted base pairing is not compensated by base pairing between guide RNA and DNA target strand. No penalty was introduced for a target mutation at position 30 due to the base flips at this position observed in the Cascade structure[9]. Resulting model energy landscapes are shown in Supplementary Fig. 14a for different numbers of terminal mismatches and applied negative torque. Elevated negative torque increases the occupancy of the full R-loops state as it becomes energetically favored, while PAM distal penalties decrease the occupancy of the full R-loop state drastically (Supplementary Fig. 14b, c). Finally, using the simplified model for $k_{lock,app}$, the measured apparent locking rate data could be globally fitted using k_{lock} and a base-pair independent mismatch penalty as fit parameters (Fig. 5, main text). Agreement between data and model supported the idea that R-loop locking only occurs for a fully extended R-loop and that the torque dependence of the $k_{lock,app}$ is mainly due to an increasing confinement of the R-loop to its full length with increasing negative supercoiling.

References

1. Sinkunas, T. *et al.* In vitro reconstitution of Cascade-mediated CRISPR immunity in Streptococcus thermophilus. *The EMBO journal* **32,** 385–394; 10.1038/emboj.2012.352 (2013).
2. Gopich, I. V. & Szabo, A. Single-molecule FRET with diffusion and conformational dynamics. *The journal of physical chemistry. B* **111,** 12925–12932; 10.1021/jp075255e (2007).
3. Bronson, J. E., Fei, J., Hofman, J. M., Gonzalez, R. L. & Wiggins, C. H. Learning rates and states from biophysical time series: a Bayesian approach to model selection and single-molecule FRET data. *Biophysical Journal* **97,** 3196–3205; 10.1016/j.bpj.2009.09.031 (2009).
4. Daldrop, P., Brutzer, H., Huhle, A., Kauert, D. J. & Seidel, R. Extending the range for force calibration in magnetic tweezers. *Biophysical Journal* **108,** 2550–2561; 10.1016/j.bpj.2015.04.011 (2015).
5. Zucchini, W., MacDonald, I. L. & Langrock, R. *Hidden Markov Models for Time Series. An introduction using R* (Chapman and Hall/CRC, Second edition / Walter Zucchini, Iain L. MacDonald, and Roland Langrock. | Boca Raton : Taylor & Francis, 2016. | Series: Monographs on statistics and applied probability, 150 | "A CRC title.", 2017).
6. Korobov, V. I. & Ochkov, V. F. Multi-Step Reactions: The Methods for Analytical Solving the Direct Problem. In *Chemical Kinetics with Mathcad and Maple* (Springer, Vienna2011), pp. 35–72.
7. Irmisch, P., Ouldridge, T. E. & Seidel, R. Modeling DNA-Strand Displacement Reactions in the Presence of Base-Pair Mismatches. *Journal of the American Chemical Society* **142,** 11451–11463; 10.1021/jacs.0c03105 (2020).
8. Rutkauskas, M. *et al.* A quantitative model for the dynamics of target recognition and off-target rejection by the CRISPR-Cas Cascade complex. *bioRxiv,* 2022.01.26.477710; 10.1101/2022.01.26.477710 (2022).
9. Mulepati, S., Héroux, A. & Bailey, S. Structural biology. Crystal structure of a CRISPR RNA-guided surveillance complex bound to a ssDNA target. *Science* **345,** 1479–1484; 10.1126/science.1256996 (2014).
