# Supplementary material for: Dynamic interplay between target search and recognition for a Type I CRISPR-Cas system
Source: Nat Commun. 2023 Jun 20;14:3654. doi: 10.1038/s41467-023-38790-1 (PMC10281945; doi:10.1038/s41467-023-38790-1)
Supplement: Supplementary file 3 — Peer Review File [file 41467_2023_38790_MOESM3_ESM.pdf]

nature portfolio

Peer Review File

Dynamic interplay between target search and recognition for a Type I CRISPR-Cas system

Open Access This file is licensed under a Creative Commons Attribution 4.0 International License, which permits use, sharing, adaptation, distribution and reproduction in any medium or format, as long as you give appropriate credit to the original author(s) and the source, provide a link to the Creative Commons license, and indicate if changes were made. In the cases where the authors are anonymous, such as is the case for the reports of anonymous peer reviewers, author attribution should be to 'Anonymous Referee' followed by a clear attribution to the source work. The images or other third party material in this file are included in the article's Creative Commons license, unless indicated otherwise in a credit line to the material. If material is not included in the article's Creative Commons license and your intended use is not permitted by statutory regulation or exceeds the permitted use, you will need to obtain permission directly from the copyright holder. To view a copy of this license, visit http://creativecommons.org/licenses/by/4.0/.

Reviewers' Comments:

Reviewer #1:
Remarks to the Author:
In their manuscript, Aldag and coauthors present a compelling study on how the CRISPR-Cas Cascade searches for it target sequences. They use single-molecule approaches (fluorescence microscopy and magnetic tweezers) in different combinations, together with advanced data analysis and well-chosen DNA substrates. They find that the target search is combination of 3D and 1D diffusion, with interesting differences with other DNA target-search processes but also simularities. Overall this is an excellent paper, great data, excellent analysis, clearly and well written, with grate visuals. I think it is well fit for the readership of Nature Comm and would be of interest to a substantial readership. I fully recommend publication. I have a couple of comments the authors might want to consider.

Fig. 4(e): the authors present an elegant and relatively simple model describing the target search. The model quantitatively describes the data pretty well, but there are some discrepancies at low torque. They devote a substantial section (from line 330) to fine tuning the model, by including R-loop locking. And this reduces the discrepancy between improved model and data, but only a bit. I think it would be good that the authors discuss what the remaining discrepancy might be due to.

I liked the discussion ll 442- in which the St-Cascade target search is compared to 'classical' ones. But to me it tasted like a bit more detail might help understanding. Could maybe an energy landscape like in 1b help in comparing the classical search mechanisms with the PAM-mediated one?

Minor points:
- l 188 I was a bit confused by "STRONGLY decreased". I think the strongly could be left out.
- l 409 why different numbers then in text?

Reviewer #2:
Remarks to the Author:
How CRISPR-Cas effectors search the target is still not fully understood and understanding the target searching mechanism is of great significance towards developing more efficient gene editing tools. In this work, the authors combined the single-molecule fluorescence and magnetic tweezers tech to visualize how the st-Cascade searches and recognizes DNA in real time, and provided a lot new information for the Cascade target searching mechanism. Overall, most experiments in this manuscript are well designed, and the major conclusion is convincing. They advanced our understanding of the Cascade target searching mechanism and will be of broad interest to the CRISPR community. I therefore suggest the publication of this work in Nature Communications if the following questions can be addressed.

Major comments
1. What is the magnetic tweezers resolution in the combined set up (1bp, 2bp…..)? The authors claimed that they saw many transient binding events without R-loops formation (page 6). Is it possible that this is because the magnetic tweezers resolution is not high enough to see the formation of R-loop (out of detection limitation). For each PAM, no matter there is a target or not, dsDNA need to unwind and perform a match test with crRNA (there are definitely transient R-loop formations during this step). The current words and conclusion could mislead people.
2. The authors claimed that Cascade forms an R-loop shortly after binding. I think this is very interesting, however, I do think they need perform more experiment to strengthen their conclusions. The instantaneous between binding and R-loop formation could also due to the resolution limitation. To rule out this, they need to provide more evidence. Maybe other substrates (6bp matches, 8bp matches….) should be tested. Also, if there are multiple AAN PAMs within this region, does the dwelling time change before R-loop formation?
3. The authors observed some transient search events in Fig S8 for both AGN and CCN PAM, however, they reported the searching efficiency for AGN and CCN PAM is zero at torque -4.7 pN nm in Figure 2g. I am confused. Why?

4. St-Cascade uses limited 1D diffusion for its target search section. Does negative torque applied in the DNA substrates? Since negative torque play an important role in target search (Figure 2f and 4e), can they study the effect of negative torque on 1D diffusion coefficient?

Minor comments
1. The EMSA in Figure S1C was confusing. I did not see EMSA for Cascade-Cy5. Where is the shift band? There is no difference between Cascade-Cy5 (control) and Cascade-Cy5-dsDNA-Cy5matching. The authors should perform a more convincing EMSA using dsDNA-Cy3matching or Cascade-Cy3.
2. Do Cas6 mutation interfere with proper assembly of the Cascade complex? The authors should show a protein SDS-PAGE gel confirming that the Cas6 mutant Cascade complexes retain the correct stoichiometry of the subunits.
3. In line 252, they claimed that tauPAM=0.5 ms. How is it calculated? I would think that the tauPAM=170/35=4.9 ms
4. Page 19, Superdex 16/60 should be 16/600.
5. line 557, 0,5 µm should be 0.5 µm.
6. Uniform references. For example ref24 and ref26

Reviewer #3:
Remarks to the Author:
In this experimental study the authors employ single-particle tracking and magnetic tweezers to unveil R-loop formation and binding of Cascade proteins in DNA. They find direct evidence for facilitated diffusion, i.e., combination of 1D and 3D search of the Cascade proteins. While I am less familiar with the biochemistry underlying this study, I mainly concentrate on the diffusive aspects & the theoretical modelling. From this perspective, I find the article to contain impressive experimental work and to deliver convincing evidence for
a specific and so-far elusive R-loop formation/stabilisation mechanism. After clarification of the minor points raised below I would expect this work to warrant publication in Nature Communications.

(1) In facilitated target search by transcription factors, it was demonstrated that the local density of DNA significantly impacts the target search rate, see Proc Natl Acad Sci USA 106, 8204 (2009). I guess this would effect significant differences between the in vitro study reported here & other setups, or the situation in vivo. This point should be briefly discussed.

It could be mentioned how the diffusion-limitation of the target search rate depends on the concentration: for 1D search it would be 1/C^2, while for pure 3D search it would be 1/C, where C is the concentration of binding agents in the bulk volume. 1D search leads to frequent revisits (recurrence in the Polya sense, often also referred to as oversampling) & thus would indeed facilitate recognition. See some discussion about the C-dependence in Biophys J 89, 895 (2005).

(2) What is the meaning of negative dwell times in the distributions shown in figure 2b? Did I miss something?

(3) Concerning the random walk model for the diffusive 1D search: I did not understand how boundaries at the two extremities of the DNA are taken into consideration. Are they included by reflective boundaries? If yes, is this justified for what is known about the interaction of the proteins with the DNA? Wouldn't they simply unbind?

Moreover, relation (S8) is only valid for unbounded motion, is this justified for the concrete scenario here? Could the authors show the mean squared displacement from simulations? What is the distribution of search times in this model, could you show a distribution of search times from your model in the SM? In this light is a mean time sufficient to characterise the search dynamics?

(4) Is it known whether the direction of encounter of the Cascade proteins with the target matters?

(5) When discussing the 1D diffusion part, a quantitative comparison with single-particle tracking for DNA-binding proteins [Phys Rev Lett 97, 048302 (2006)] could be helpful to judge the mobility of the Cascade proteins.

Leipzig, March 21st 2023

Point by Point response to the reviewers

We thank all reviewers for their constructive and detailed comments. We hope that the revised version satisfies their requests and is now suited for publication.

Reviewer #1 (Remarks to the Author):

In their manuscript, Aldag and coauthors present a compelling study on how the CRISPR-Cas Cascade searches for it target sequences. They use single-molecule approaches (fluorescence microscopy and magnetic tweezers) in different combinations, together with advanced data analysis and well-chosen DNA substrates. They find that the target search is combination of 3D and 1D diffusion, with interesting differences with other DNA target-search processes but also simularities. Overall this is an excellent paper, great data, excellent analysis, clearly and well written, with grate visuals. I think it is well fit for the readership of Nature Comm and would be of interest to a substantial readership. I fully recommend publication. I have a couple of comments the authors might want to consider.

Point by point reply to Reviewer #1:

- Fig. 4(e): the authors present an elegant and relatively simple model describing the target search. The model quantitatively describes the data pretty well, but there are some discrepancies at low torque. They devote a substantial section (from line 330) to fine tuning the model, by including R-loop locking. And this reduces the discrepancy between improved model and data, but only a bit. I think it would be good that the authors discuss what the remaining discrepancy might be due to.

  Response: We added a paragraph to the discussion acknowledging this fact and offering some further explanation (from l 467).

- I liked the discussion ll 442- in which the St-Cascade target search is compared to 'classical' ones. But to me it tasted like a bit more detail might help understanding. Could maybe an energy landscape like in 1b help in comparing the classical search mechanisms with the PAM-mediated one?

  Response: For target recognition, we have a slow, sequential, multi-step process with defined 1-bp precision energy wells. This is not true for the target search where the nature of the reaction coordinate would remain unclear, making the designing of an energy landscape for the target search complex. However, we rephrased this part in the manuscript and added a schematic representation of the comparison to the SI (now Supplementary Fig. 15) and hope to have thereby made the point clear to a satisfactory degree (ll 442).

Minor points:

- l 188 I was a bit confused by "STRONGLY decreased". I think the strongly could be left out.

  Response: We have removed the word “strongly” in the manuscript.

- l 409 why different numbers then in text?

  Response: We assume that what is meant is a difference in the numbers between l 409 and Fig. 5 and its caption. We have adjusted the numbers in the text.

Reviewer #2 (Remarks to the Author):

How CRISPR-Cas effectors search the target is still not fully understood and understanding the target searching mechanism is of great significance towards developing more efficient gene editing tools. In this work, the authors combined the single-molecule fluorescence and magnetic tweezers tech to visualize how the st-Cascade searches and recognizes DNA in real time, and provided a lot new information for the Cascade target searching mechanism. Overall, most experiments in this manuscript are well designed, and the major conclusion is convincing. They advanced our understanding of the Cascade target searching mechanism and will be of broad interest to the CRISPR community. I therefore suggest the publication of this work in Nature Communications if the following questions can be addressed.

Point by point reply to Reviewer #2:

Major comments

- What is the magnetic tweezers resolution in the combined set up (1bp, 2bp…..)? The authors claimed that they saw many transient binding events without R-loops formation (page 6). Is it possible that this is because the magnetic tweezers resolution is not high enough to see the formation of R-loop (out of detection limitation). For each PAM, no matter there is a target or not, dsDNA need to unwind and perform a match test with crRNA (there are definitely transient R-loop formations during this step). The current words and conclusion could mislead people.

  Response: The experimental setup used here cannot detect dynamic R-loop formation on a bp level. For that, a different assay is needed. However, in our experiments, we define a successful target search by a stable R-loop formation. The stability is measured by the duration of the formed R-loop. In our experiments, a stable R-loop lasts, on average, ~10s, as mentioned in line 123. This is well above the resolution limit of our experimental setup, as thoroughly described in Supplementary Methods 1. Unstable R-loops, as described by the reviewer, are by definition part of an unsuccessful target search, even if performed at the correct location/PAM. One of the main conclusions of our work is that finding the correct PAM is not necessarily followed by target recognition (stable R-loop formation). We slightly modified our wording in line 176 and 177 of the manuscript to make the point clearer.

- The authors claimed that Cascade forms an R-loop shortly after binding. I think this is very interesting, however, I do think they need perform more experiment to strengthen their conclusions. The instantaneous between binding and R-loop formation could also due to the resolution limitation. To rule out this, they need to provide more evidence. Maybe other substrates (6bp matches, 8bp matches….) should be tested. Also, if there are multiple AAN PAMs within this region, does the dwelling time change before R-loop formation?

  Response: R-loop formation does not occur instantaneously after binding but within less than 100ms. In Supplementary Methods 2, we explain in detail how we determine the dwell time from DNA binding to R-loop formation and how we take the resolution of our experimental setup into account. We also describe the use of simulated traces with short-lived events to

reinforce our analysis. We believe that, given the number of observed events (N=173), our results are robust. From other experiments, we know that R-loop formation itself happens on a scale of 10ms for 26bp R-loops (Kauert et al., 2023, see revised manuscript). Given the reduced length of the R-loop in the presented work (12bp), we believe that R-loop formation itself has no significance within error on the measured dwell times. Therefore, we do not believe that additional experiments with modified R-loop lengths would reveal any further insights. We added this explanation with the citation to the revised manuscript (from l 170).

Regarding the number of PAMs, it is already high, given its generic nature (AAN). On average, there is a cognate AAN-PAM every 8bp. Engineering of a substrate with no or very few PAMs is highly difficult given the long diffusion lengths of the protein complex. However, we successfully show that we can modulate the dwell time by changing the applied torque or leaving out the target.

- The authors observed some transient search events in Fig S8 for both AGN and CCN PAM, however, they reported the searching efficiency for AGN and CCN PAM is zero at torque -4.7 pN nm in Figure 2g. I am confused. Why?

  Response: The search efficiency is defined by the number of productive binding events with successful (stable) R-loop formation divided by the total number of binding events (including the transient, non-productive ones). Since there were no successful binding events for these PAMs, the search efficiency was zero.

- St-Cascade uses limited 1D diffusion for its target search section. Does negative torque applied in the DNA substrates? Since negative torque play an important role in target search (Figure 2f and 4e), can they study the effect of negative torque on 1D diffusion coefficient?

  Response: With our cylindrical magnet configuration (lateral stretching of DNA), the application of torque is unfortunately not possible. However, our experiments show that the search times of non-productive events at -4.7pN nm for CC and AG PAMs match, within error, the results at 0 pN nm for the canonic AAN PAM (Fig 2f). This indicates that the diffusion itself is not strongly affected by torque. As we describe in the manuscript, the reduced dwell times stem from facilitated target recognition/R-loop formation. We believe that any further study of this topic is beyond the scope of the presented study. We found one study showing the dependence of 1D diffusion on supercoiling (https://doi.org/10.1073/pnas.1908826116). However, these effects were shown at considerably higher negative torques than were used in our experiments. At these torques, DNA melting and other DNA structures appear, which do not appear at the levels of torque used in our experiments. We added a sentence and the source of the paper to the manuscript (l. 432).

Minor comments

- The EMSA in Figure S1C was confusing. I did not see EMSA for Cascade-Cy5. Where is the shift band? There is no difference between Cascade-Cy5 (control) and Cascade-Cy5-dsDNA-Cy5matching. The authors should perform a more convincing EMSA using dsDNA-Cy3matching

or Cascade-Cy3.

Response: We repeated the EMSA and added it to a new version of the Supplementary Information (Supplementary Figure 1).

- Do Cas6 mutation interfere with proper assembly of the Cascade complex? The authors should show a protein SDS-PAGE gel confirming that the Cas6 mutant Cascade complexes retain the correct stoichiometry of the subunits.

  Response: The SDS-Page gel is displayed in Supplementary Figure 15a (16a in the revised SI). It can be seen there that Cas8e is missing from the complex after purification. As is explained in the Methods ("Expression and purification of proteins"), it is therefore independently expressed and subsequently added to the complex, regaining activity as shown in the activity assay in Supplementary Figure 15b.

- In line 252, they claimed that tauPAM=0.5 ms. How is it calculated? I would think that the tauPAM=170/35=4.9 ms

  Response: The complex does not visit each PAM only once. In a random walk, the mean dwell time per step is calculated by $\Delta t = \frac{\Delta^2}{D}$, with Δ being the mean step distance and D being the diffusion constant (see Supplementary Methods 4 for details).

- 4. Page 19, Superdex 16/60 should be 16/600.

  Response: This was corrected in the manuscript.

- line 557, 0,5 µm should be 0.5 µm.

  Response: This has been corrected in the manuscript.

- Uniform references. For example ref24 and ref26

  Response: This has been corrected in the manuscript.

Reviewer #3 (Remarks to the Author):

In this experimental study the authors employ single-particle tracking and magnetic tweezers to unveil R-loop formation and binding of Cascade proteins in DNA. They find direct evidence for facilitated diffusion, i.e., combination of 1D and 3D search of the Cascade proteins. While I am less familiar with the biochemistry underlying this study, I mainly concentrate on the diffusive aspects & the theoretical modelling. From this perspective, I find the article to contain impressive experimental work and to deliver convincing evidence for
a specific and so-far elusive R-loop formation/stabilisation mechanism. After clarification of the minor points raised below I would expect this work to warrant publication in Nature Communications.

Point by point reply to Reviewer #3:

- In facilitated target search by transcription factors, it was demonstrated that the local density of DNA significantly impacts the target search rate, see Proc Natl Acad Sci USA 106, 8204 (2009). I guess this would effect significant differences between the in vitro study reported here & other setups, or the situation in vivo. This point should be briefly discussed.

  Response: We have added a segment in the discussion along with a citation of the mentioned publication (from l 440).

- It could be mentioned how the diffusion-limitation of the target search rate depends on the concentration: for 1D search it would be 1/C^2, while for pure 3D search it would be 1/C, where C is the concentration of binding agents in the bulk volume. 1D search leads to frequent revisits (recurrence in the Polya sense, often also referred to as oversampling) & thus would indeed facilitate recognition. See some discussion about the C-dependence in Biophys J 89, 895 (2005).

  Response: We added a paragraph discussing the salt dependence and added the source (From l 440).

- (2) What is the meaning of negative dwell times in the distributions shown in figure 2b? Did I miss something?

  Response: The negative dwell times are a consequence of the resolution limit of our experimental setup. We consider the recorded dwell time distributions as a convolution of an exponential (the actual dwell time) and a normal distribution (the noise). This is explained in detail in the Supplementary Methods 1 and 2.

- (3) Concerning the random walk model for the diffusive 1D search: I did not understand how boundaries at the two extremities of the DNA are taken into consideration. Are they included by reflective boundaries? If yes, is this justified for what is known about the interaction of the proteins with the DNA? Wouldn't they simply unbind?

  Response: Both boundaries in our model are considered to be reflective. In our experiments, one end of the DNA is limited by the surface of the flowcell, as the DNA is attached to it. On the

other side of the area imaged by the TIRF microscope, the boundary is not truly reflective. However, given that proteins can diffuse back into the imaged area (random walk), we believe that the approximation of a reflective boundary is close enough. We added a sentence to the Supplementary Methods 4 to point out the reflective nature of the boundaries (l 255).

- Moreover, relation (S8) is only valid for unbounded motion, is this justified for the concrete scenario here? Could the authors show the mean squared displacement from simulations?

  Response: Relation (S8) is used to infer the stepping rate of the Cascade complex during the target search from our lateral diffusion measurements. Given that the DNA substrate used in the experiment is approximately 5µm (15kbp) long and the Cascade complex scans on average a distance of 90nm, we believe that the assumption of unbounded diffusion holds. We added a few sentences to the SI to make this point clearer (l 260).

- What is the distribution of search times in this model, could you show a distribution of search times from your model in the SM? In this light is a mean time sufficient to characterise the search dynamics?

  The search times from our model are distributed exponentially (see image below). The mean time in an exponential distribution is a significant description parameter of that distribution.

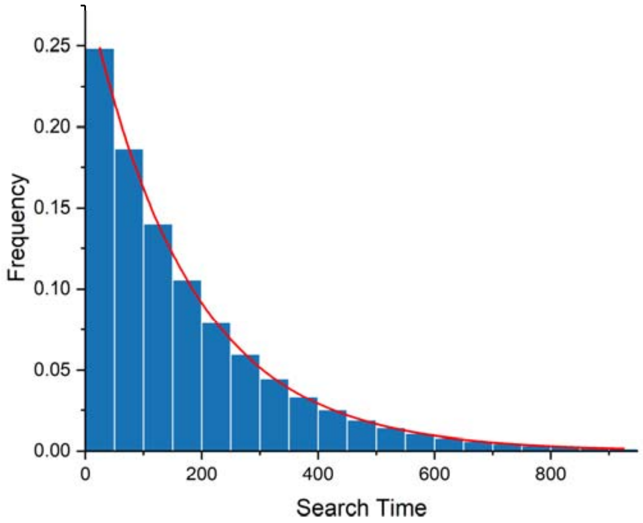

Figure: Dwell time distribution from target search model. Blue bars show dwell time distribution. Red line depicts exponential fit.

- (4) Is it known whether the direction of encounter of the Cascade proteins with the target matters?

  Response: Unfortunately, this is not known.

- (5) When discussing the 1D diffusion part, a quantitative comparison with single-particle tracking for DNA-binding proteins [Phys Rev Lett 97, 048302 (2006)] could be helpful to judge the mobility of the Cascade proteins.

  Response: We added a sentence to the manuscript discussing the mobility of St-Cascade compared to other DNA-binding proteins and added the proposed citation (l 442).

Reviewers' Comments:

Reviewer #1:
Remarks to the Author:
In the revised version of their manuscript, Aldag et al. have addressed the (small) questions I had on the earlier version satisfactory. I wholeheartedly support publication of this great and well-written story in its current form.

Reviewer #2:
None

Reviewer #3:
Remarks to the Author:
I am happy with the responses to the points raised in my report. This is a high quality study, and I warmly recommend publication of this revised version in Nature Communications.
